# Incorporation of branched-chain fatty acid into cellular lipids and caspase-independent apoptosis in human breast cancer cell line, SKBR-3

**DOI:** 10.1186/1476-511X-4-29

**Published:** 2005-11-23

**Authors:** Sawitree Wongtangtintharn, Hirosuke Oku, Hironori Iwasaki, Masashi Inafuku, Takayoshi Toda, Teruyoshi Yanagita

**Affiliations:** 1 United Graduate School of Agricultural Sciences, Kagoshima University, 1-21-24, Korimoto, Kagoshima 890-0065, Japan; 2 Center of Molecular Bioscience, University of the Ryukyus, Nishihara, Okinawa 903-0213, Japan; 3 Department of Clinical Laboratory Medicine, University of the Ryukyus Hospital, School of Medicine, Uehara 207, Nishihara, Okinawa 903-0125, Japan; 4 Department of Applied Biological Sciences, Saga University, Saga 840-8502, Japan

**Keywords:** branched-chain fatty acid, incorporation, glycerolipid, apoptosis, caspase-independent

## Abstract

**Background:**

13-Methyltetradecanoic acid (13-MTD), an iso-C15 branched- chain saturated fatty acid, has been shown to induce apoptotic cell death of numerous human cancer cells. However, the mechanism for the induction of apoptosis has not been fully understood. This study described the incorporation of 13-MTD into cellular lipid of SKBR-3 breast cancer cells and apoptosis related event to gain more insight into the mechanism action of this fatty acid.

**Results:**

Treatment of SKBR-3 cells with 13-MTD lowered the cell viability and induced apoptosis. Proportion of 13-MTD in the glycerolipids increased to saturation level within 6 hours. Triacylglycerol contained 13-MTD in higher concentration than phospholipid with positional preference to sn-2. 13-MTD caused no changes in the caspase activity and its gene expression. Furthermore, addition of caspase-inhibitor to culture medium did not prevent the cells from the cytotoxicity of 13-MTD. No-increase in the cellular calcium level was also noted with 13-MTD treatment. However, 13-MTD disrupted the mitochondrial integrity in 4 hours, and increased the nuclear translocation of apoptosis inducing factor.

**Conclusion:**

These results showed that 13-MTD disrupted the mitochondrial integrity, and induced apoptosis via caspase-independent death pathway.

## Background

The branched-chain fatty acid (BCFA) or alcohols has been reported to suppress the proliferation or development of tumor cells [3]. Recently, 13-methyltetradecanoic acid (13-MTD), an iso-C15 saturated fatty acid, was purified as an anti-cancer agent from a soy-fermentation product [4]. 13-MTD inhibited cell proliferation and induced apoptotic cell death in many cancer cells such as prostate carcinoma (DU-145), leukemia (K563) and mammary adenocarcinoma (MCF-7) [4].

Induction of apoptosis was characterized by many morphological features including membrane blebbing, cytoplasmic and nuclear condensation, DNA fragmentation and formation of apoptotic bodies [5,6]. Our previous study showed that 13-MTD synthetically lowered the fatty acids biosynthesis and reduced the precursor of free fatty acid for phospholipid synthesis in human breast cancer cell (SKBR-3) [3]. However, the mechanism for the induction of apoptosis by 13-MTD has not been fully understood.

Epidemiological studies have indicated that fatty acid composition of the diet correlated with the risk of mammary cancer [7-9]. The level of stearic acid in tumor membrane phosphatidylcholine has been considered to be an independent tumor marker of breast cancer prognosis [10]. Furthermore, the involvement of lipogenic enzyme in oncogenesis has suggested that the membrane lipids could be a factor for the modulation of tumor growth [11,12]. It is therefore possible that the incorporation of 13-MTD into membrane or storage lipid may play a role for the induction of apoptosis in the cancer cells. Thus the aim of this study was to describe the incorporation of 13-MTD into cellular glycerolipid and its positional distribution in the lipid molecules. Furthermore, this study shed the light on the effect of 13-MTD on apoptosis related events to gain more insight into the mechanism actions of this fatty acid.

## Results

**Figure 1:**
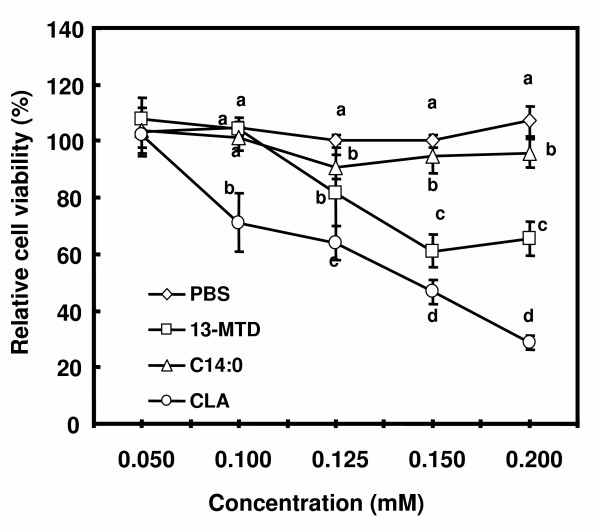
Effect of 13-MTD on the relative viability of SKBR-3 cells. Cells were treated with the fatty acid samples for 24 h, and the relative viabilities were determined by MTS assay kit according to the manufacture's instruction. Data are mean ± SE of 8 analyses. Data not sharing the same letter within each concentration are significantly different at p < 0.05 by Tukey-Kramer test.

**Figure 2:**
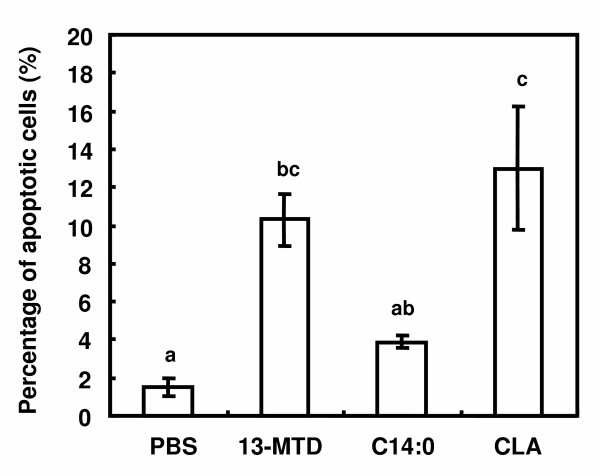
Effect of 13-MTD on the induction of apoptotic cell death in SKBR-3 cells. Cells were pre-cultured for 24 h and treated with the fatty acid samples for another 4 h, and apoptotic cells were measured by Anexin V staining. Number of Anexin V stained cells in approximately 100 cells was counted and expressed as percentages of apoptotic cells. Data are mean ± SE of 4 analyses. Data not sharing the same letter shows statistically significant difference at p < 0.05 by Tukey-Kramer test.

The effect of 13-MTD on the relative viability of SKBR-3 cells are shown in figure 1. Consistent with the previous results, 13-MTD lowered the cell viability compared with the straight chain fatty acid counterpart, but to a lesser extent than *cis*-9, *trans*-11 conjugated linoleic acid (CLA), a well known anticancer fatty acid. Quantitation of apoptotic cells found that 13-MTD as well as CLA increased the number of apoptotic cells even after treatment of 4 hours (Fig. 2).

**Figure 3:**
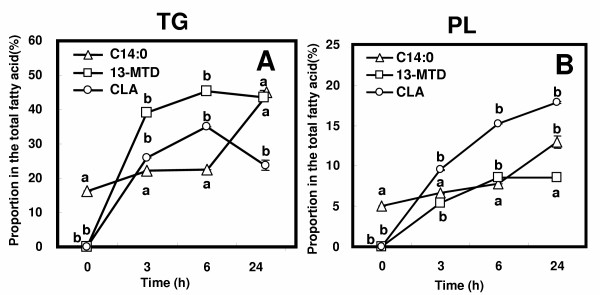
Time course of 13-MTD incorporation into triacylglycerol (A) and phospholipids (B) of SKBR-3 cells. SKBR-3 cells grown to 80% confluency were incubated with the fatty acid samples for 3 to 24 h, and their proportions in the total fatty acids were measured by gas-chromatograph. Data are mean ± SE of triplicate analyses. Data not sharing the same letter shows statistically significant difference at p < 0.05 by Tukey-Kramer test.

Figure 3 illustrates the incorporation of 13-MTD, CLA and myristic acid into cellular glycerolipid, TG (A) and PL (B). Proportions of 13-MTD and CLA in both PL and TG increased with the incubation time. 13-MTD was incorporated preferentially into TG rather than PL. The proportion of 13-MTD in both PL and TG increased to the saturation level in 6 h. As was the case of 13-MTD, preference for TG was noted with the incorporation of CLA into cellular lipid. However, the proportion of CLA in PL was much higher than that of 13-MTD, showing that CLA accumulated in membrane lipid to a greater extent than 13-MTD.

**Figure 4:**
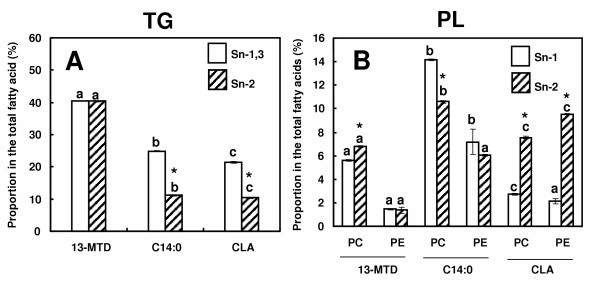
Positional distribution of 13-MTD in triacylglycerol (A) and phospholipids (B) of SKBR-3 cells. Cells were grown to 80% confluency, and incubated with the fatty acid samples for 6 hours. Phospholipid and triacylglycerol fractions were hydrolyzed respectively with lipase and phospholipase A2, and positional distribution of sample fatty acid was determined as described in the method section. Data are mean ± SE of triplicate analyses. Data not sharing the same letter shows statistically significant difference at p < 0.05 by Tukey-Kramer test. Asterisk shows the statistical significance between position 1,3 and position 2 by Student's t-test.

Fatty acids taken up by the cells undergo esterification to form membrane PL or storage TG, depending probably on their chemical structure. The metabolic fate of fatty acid thus showed diversity with their chemical structure, and resulted in uneven positional distribution in TG or PL molecules [17]. TG contained almost equal proportions of 13-MTD at sn-2 position and at sn-1,3 positions (Fig 4A). However, this was not the case for CLA: sum of sn-1 and sn-3 position was higher than the proportion at sn-2 position.

Preferential esterification of 13-MTD to sn-2 position was noted for PC, while this trend was more clearly shown for the case of CLA (Fig. 4B). Incorporation of 13-MTD into PC was higher than that into PE. CLA distributed evenly between PC and PE.

**Figure 5:**
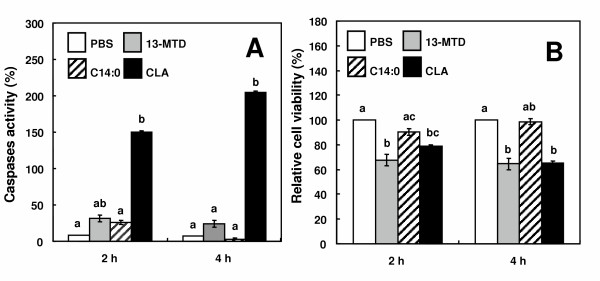
Effect of 13-MTD on caspase-3 activity (A) and relative viability (B) of SKBR-3 cells. Cells were incubated with the fatty acid samples (0.25 mM) for 2 or 4 h. Caspase activity in the cell lysate and the cell viability were determined as described in the method section. Data are mean ± SE of triplicate analyses. Data not sharing the same letter shows statistically significant difference at p < 0.05 by Tukey-Kramer test.

Caspases are involved in signal transduction leading to apoptotic cell death [18,19]. Caspase 3 acts at the final step of caspase cascade to cleave proteins involved in cytoskeletal and nuclear structure, resulting in the cell shrinking and DNA fragmentation of late-stage apoptosis. 13-MTD has been demonstrated to be an anticancer agent to induce apoptosis in a variety of cancer cells. However, 13-MTD had no effect on the activity of caspase 3 in SKBR-3 cells. (Fig. 5A) In contrast to 13-MTD, CLA significantly elevated the caspase 3 activity after 2 and 4 h of incubation. Despite lack of caspase 3 activation, 13-MTD reduced the cell viability to the same extent as CLA did (Fig. 5B).

**Figure 6:**
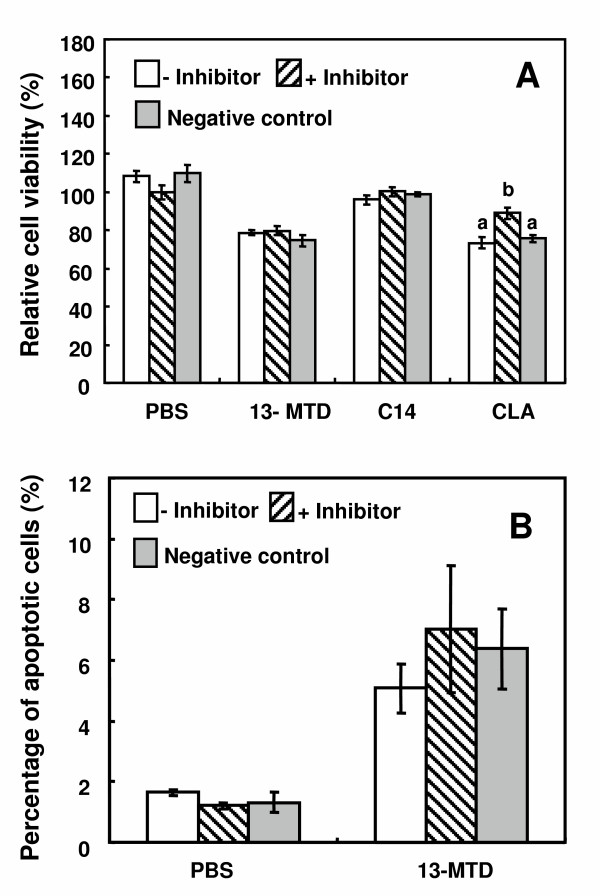
Effect of caspase-3 inhibitor on cytotoxicity (A) and induction of apoptosis (B) by13-MTD. Cells pre-cultured for 24 h were incubated with PBS (no inhibitor), caspase-3 inhibitor (20 *μ*M Z-DEVD-FMK) and negative control inhibitor (20 *μ*M Z-FA-FMK) for 60 min and then further incubated with 0.25 mM of fatty acids samples for 4 h. On completion of the incubation, cell viability was determined by MTS assay (A), and induction of apoptosis was assayed as described in legend to Fig. 2 (B). Data are mean ± SE of triplicates analyses. Data not sharing the same letter within the treatment shows the statistically significant difference at p < 0.05 by Tukey-Kramer test.

To confirm the non-involvement of caspase pathway, cytotoxicity and apoptosis induction were studied in the presence of caspase 3 inhibitor (Z-DEVD-FMK) and negative control for caspase-3 inhibitor (Z-FA-FMK). The negative control (Z-FA-FMK) only inhibits cysteine protease, and has no inhibitory effect on apoptosis mediated by caspase. Presence of caspase-3 inhibitor did not alleviate the cytotoxicity of 13-MTD (Fig. 6A), and also did not prevent the cells from induction of apoptosis (Fig. 6B). However, presence of the caspase inhibitor, but not the negative control, almost abolished the cytotoxicity of CLA (Fig. 6A).

**Figure 7:**
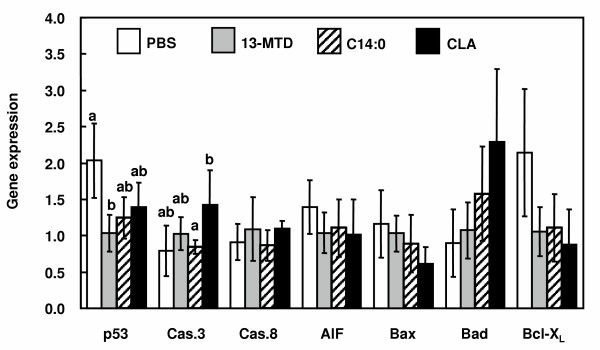
Effect of 13-MTD on the expression of several regulatory genes involved in the apoptosis induction in SKBR-3 cells. Cells pre-cultured for 24 hours were treated with the fatty acids for 4 h, and gene expression were measure by real time PCR. The relative change in gene expression was quantified essentially based on the 2 ^-ΔΔCT ^methods. Data are mean ± 95% confidence intervals of triplicates analyses. Data not sharing the same letter shows the statistically significant difference at p < 0.05 by Tukey-Kramer test.

Effect of 13-MTD on the expression of several regulatory genes involved in the apoptosis induction was studied (data not shown). Addition of fatty acid samples evenly decreased the expression of p53 gene with no difference between 13-MTD and myristic acid treatment. CLA increased mRNA level of caspase 3 as well as its activity. 13-MTD had no effect on the expression of caspase 8, apoptosis inducing factor (AIF), Bax, Bad and Bcl-XL (Fig. 7).

**Figure 8:**
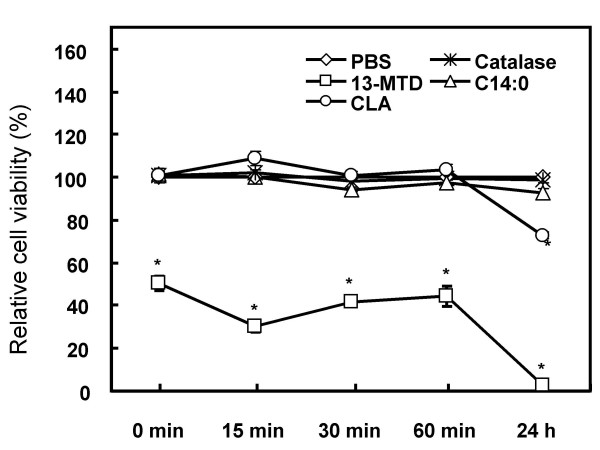
Cytotoxicity of 13-MTD to SKBR-3 cells in the presence or absence of catalase. Cells were pre-cultured for 24 h. Catalase (100 units/ml) was added at indicated time of incubation with 0.25 mM of fatty acid samples, and the relative cell viability was measured after 24 h by MTS assay. Data are mean ± SE of 8 analyses. Asterisk shows the statistically significant difference from PBS or C14:0 at p < 0.05 by Tukey-Kramer test.

It has been demonstrated that hydrogen peroxide mediates the induction of apoptosis in response to several external stimuli [20,21], and the supplementation of the medium with catalase prevent the cells from apoptosis induction [22,23]. Addition of catalase to the culture medium did not protect or rescue the cells from cytotoxicity of 13-MTD (Fig. 8). In agreement with the previous result, presence of catalase in the culture medium protected the cells from the cytotoxicity of CLA (Fig. 8).

**Figure 9:**
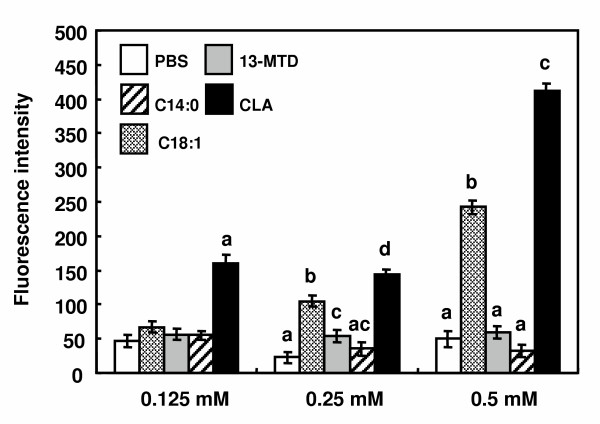
Effect of 13-MTD on cellular calcium mobilization in SKBR-3 cells. SKBR-3 cells were seeded into black 96-well plate at 10^5 ^cells/well, and incubated for 1 day. Cells were loaded with 4 μM Fluo 3-AM for 30 min, and subsequently stimulated with 0.125, 0.25 and 0.5 mM fatty acids in 0.8% Tween 80 in PBS. The increases in fluorescence were measured 25 times with 0.15 s intervals. Data are mean ± SE of 38 replicate analyses. Data not sharing the same letter are significantly different at p < 0.05 by Tukey-Kramer test.

Several lines of studies have demonstrated that short chain or medium chain fatty acid increased the cellular Ca^2+ ^mobilization via G-protein coupled orphan receptors [24,25]. Furthermore, excess loading of mitochondria with Ca^2+ ^results in abnormal mitochondrial metabolism, which triggers the programmed cell death [26]. No mobilization of Ca^2+ ^occurred when the cells were loaded with 13-MTD (Fig. 9). Oleic acid included as positive control stimulated the cellular Ca^2+ ^mobilization as well as the case for CLA (Fig. 9).

**Figure 10:**
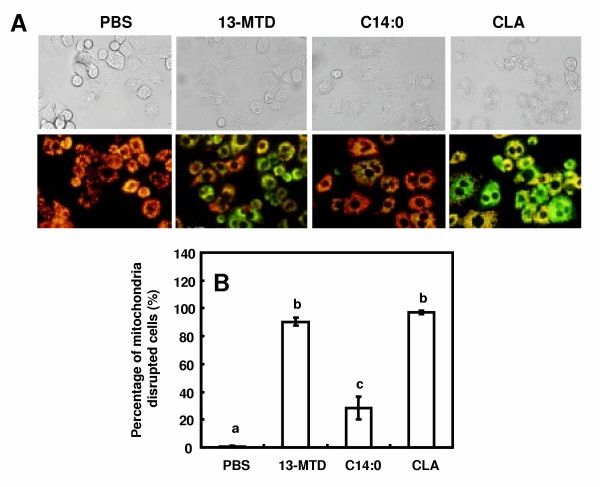
Effect of 13-MTD on mitochondrial membrane integrity of SKBR-3 cells. Cells cultured for 24 h on coverlips were incubated with 0.25 mM fatty acid sample for 4 h, and loaded with MitoCapture (Biovision, USA) reagent for 20 min at 37°C under 5% CO_2 _atmosphere. Cells were observed with a fluorescence microscope using band-pass filter. MitoCapture aggregates and fluoresces red in the mitochondria of healthy cells. However, in apoptotic cells, MitoCapture cannot accumulate in the mitochondria, it remains as monomers in the cytoplasm, and fluoresces green. Counting of mitochondria disrupted cells in total number of 40 cells were made for 4 to 6 separate microscopic fields, and the percentages are shown (B). Data not sharing the same letter are significantly different at p < 0.05 by Tukey-Kramer test.

As to induction of apoptosis, mitochondria play a pivotal role in the signal transduction pathways [27]. To examine mitochondrial membrane integrity, MitoCapture reagent was used to stain the apoptotic cells after treatment of the cells with 13-MTD. In healthy cells, MitoCapture accumulates and aggregates in the mitochondria, and gives off a bright red fluorescence, while in apoptotic cells, MitoCapture can not enter mitochondria due to altered mitochondrial transmembrane potential, and therefore remain predominantly in the cytosol in its monomer form fluorescing green. After 4 hours of treatments with 13-MTD, the MitoCapture dye fluoresced predominantly green was indicating the disruption of mitochondrial integrity (Fig. 10). Myristic acid slightly increased the staining of green fluorescence compared with control vehicle buffer, but to much lower extent than 13-MTD did. CLA treatment for 4 hours liberated the cells from the bottom of the culture dishes into medium, and resulted in staining green fluorescence (Fig. 10).

**Figure 11:**
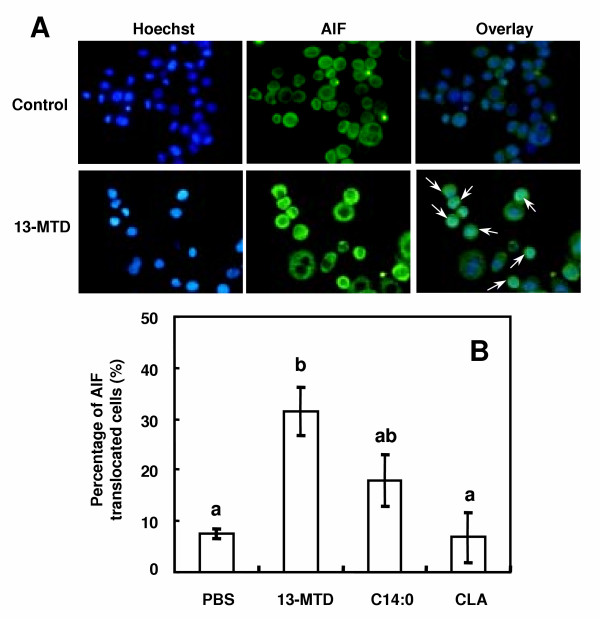
Effect of 13-MTD on nuclear translocation of AIF in SKBR-3 cells. Cells were grown for 24 h on coverslips and treated with 0.25 mM fatty acid samples for 6 h. After the treatment, cells were stained with Hoechst 33258 and AIF as described in the method section (A). Counting of AIF punctuated nuclei in total number of 40 cells were made for 4 to 6 separate microscopic fields, and the percentages of AIF translocated cells are shown (B). Data not sharing the same letter are significantly different at p < 0.05 by Tukey-Kramer test. Arrow shows the AIF translocated nucleus.

The most notorious apoptotic factors released from permeabilized mitochondria are cytochrome C and AIF. However, the results shown in the foregoing studies may rule out the involvement of cytochrome C in the death pathway of 13-MTD induced apoptosis. AIF was therefore the most plausible candidate of death signal transducer for 13-MTD induced apoptosis. Figure 11 shows the immunodetection of AIF punctuated apoptotic nucleus after treatment of the cells with 13-MTD. Treatment of the cells with vehicle buffer (control) showed few nuclear staining of AIF (Fig. 11). However, the percentage of AIF punctuated apoptotic nuclei was significantly increased by 13-MTD treatment (Fig. 11). This percentage was increased to some extent by the treatment of myristic acid. However the difference between control and myristic acid was statistically insignificant.

## Discussion

This study described the incorporation of 13-MTD into cellular lipid, and the apoptosis related events after treatment of the cells with this fatty acid. 13-MTD induced apoptosis after 4 hours of treatment (Fig. 2), and reduced the cell viability (Fig. 1). The cytotoxicity of 13-MTD may be challenged by a claim that its toxic effect is due to non-specific detergent properties of fatty acids [28]. However, myristic acid, the straight chain fatty acid counterpart, had no significant cytotoxicity against SKBR-3 cells. Furthermore, the detergent property of 13-MTD may have limited significance in the current experiment because the sample fatty acids were dissolved in a detergent buffer containing 0.8% Tween 80. The vehicle buffer (PBS containing 0.8%Ttween 80) displayed negligible effect on the cell viability (data not shown). These observations negate the possibility that the cytotoxicity of 13-MTD is due to non-specific detergent properties of 13-MTD.

Structural membrane glycerolipids of animal cells contain almost exclusively 14 to 22 carbon fatty acids of straight chain with up to three methylene-interrupted double bonds. These fatty acids were referred to as usual fatty acids. Contrary to these fatty acids, 13-MTD is a fatty acid with branched carbon chain, and can be classified as unusual fatty acid.

Fatty acid entered the cancer cells undergo esterification to form TG or PL, and the production of TG and PL shares a common pathway referred to as the glycerol-3-phosphate pathway. In plant cells, unusual fatty acids are sequestered into storage lipid TG, and have no structural functions [29]. In current study, the concentration of 13-MTD in TG was higher than that found in PL. This observation may favor the above view that the fatty acids of which chemical structure deviate from the common fatty acids are edited out from membrane PL, and channeled into TG (Fig. 3). The targeting of 13-MTD to TG may be primarily explained by the substrate specificity of the enzyme involved in the biosynthesis of PL and TG: thioesterase, acyltransferase and cytidine diphosphocholine diacylglycerol choline phosphotransferase (CDP-CPT). Of the enzymes, CDP-CPT catalyzes the reversible conversion of DG to PC. Therefore, this enzyme could potentially prevent DG species containing 13-MTD from moving into phospholipid pool. Thus it become of interest to clarify the biological machinery to channel 13-MTD into storage lipid pool.

CLA known as anticancer fatty acid was also preferentially incorporated into TG as well as the case for 13-MTD. However, PL contained much higher concentration of CLA compared with 13-MTD, suggesting that CLA rather than MTD was more compatible with the membrane structure. Conversely, PL containing 13-MTD may not be acceptable to membrane structure, and for this reason the large proportion of 13-MTD may be edited out to accumulate as TG. In this context, it is noteworthy that the membrane fluidity is a factor to regulate the activities of membrane-bound enzymes [30,31]. Thus it is plausible that 13-MTD incorporated into PL affects the mitochondrial membrane fluidity because of its branching chain structure, and hence modulates the enzyme activities leading to abnormal mitochondrial metabolism.

Positional distribution of 13-MTD also showed its characteristics in the preference toward positon-2. In the case of TG, sum proportion of 13-MTD at position 1 and 3 was almost equal to that at position 2, showing that the individual proportion at position 1 or 3 was lower than that at position 2 (Fig. 4). Same trend in the positional distribution was also seen with the case of structural lipid PC. This characteristic also may be explained by the enzyme specificity involved in the biosynthesis of these lipids.

This study reinforced the cytotoxicity of 13-MTD. 13-MTD, as well as the known anticarcinogenic fatty acid CLA, induced apoptotic cell death of breast cancer cells. Several lines of studies demonstrated that CLA induced apoptosis via caspase-dependent mechanism [29,32-34]. However, the mechanism action of 13-MTD differed from that of CLA. Incubation of the cells with 13-MTD induced no changes in the caspase activity and its gene expression (Figs. 5 and 7). Furthermore, inclusion of caspase-3 inhibitor in the culture medium did not prevent the cells from the cytotoxicity of 13-MTD (Fig. 6). These findings suggested that 13-MTD induced apoptosis via caspase-independent signal transduction.

The mitochondria are playing central role in both caspase-dependent and caspase-independent death pathway [26,35]. Mitochondria respond to multiple death stimuli which induce mitochondrial membrane permeabilization, and cause the release of apoptotic molecules such as cytochrome C or apoptosis inducing factor (AIF). Incubation of the breast cancer cells for 4 h with 13-MTD caused disruption of mitochondrial integrity (Fig. 10). This observation suggested that 13-MTD permeabilized the mitochondrial membrane, and proapoptotic molecules were released from mitochondria to trigger the chromatin condensation. These proapoptotic molecules can also be released from mitochondria by cellular perturbations that cause a sudden increase in intracellular calcium level [25]. However, 13-MTD induced no mobilization of calcium hence no increase in the intracellular calcium concentration (Fig. 9). Thus 13-MTD appeared to induce apoptosis without changing cellular calcium level.

The most notorious apoptotic factors released from permeabilized mitochondria are cytochrome C and AIF. However, the results shown in this study (Figs. 5-6) may rule out the involvement of cytochrome C in the death pathway of 13-MTD induced apoptosis. AIF was therefore the most plausible candidate of death signal transducer for 13-MTD induced apoptosis. Immunocytochemical analysis supported this view. The percentage of AIF punctuated apoptotic nuclei was increased by 13-MTD treatment (Fig. 11), suggesting that nuclear AIF translocation could be the main death signaling system. More recently, it has been demonstrated that permeabilized mitochondria can also release other proapoptotic factors such as Smac/Diablo [36,37] or HtrA2/Omi [38-40]. Involvement of these factors in the action mechanisms of 13-MTD should be explored in the coming study.

AIF is usually present in the intermembrane space of mitochondria, and translocated from mitochondria to nucleus to initiate apoptosis [41]. In most cases of apoptotic cell death, the release of AIF appeared to coincide with the loss of mitochondrial membrane integrity [42-44]. Permeabilization of mitochondrial membrane triggers the death pathway by releasing the proapoptotic molecules. The liberation mode and rate of these molecules appeared to depend on the death stimulus [44,45]. In the case of 13-MTD treatment, translocation of AIF may precede the release of cytochrome C, and play a crucial role in the progress of cell death. AIF translocation after 13-MTD treatment may be accompanied by the release of cytochrome C. However, this event usually occurs during the end stage of cell death, and has been considered to be limited significance for the completion of apoptosis [46].

13-MTD differed from CLA in that this unusual fatty acid preferentially induced translocation of AIF rather than release of cytochrome C. This feature may bear relevance with its chemical structure which appeared to be less tolerable to the membrane structure than CLA. Phospholipids isolated from 13-MTD treated cells contained 13-MTD to some extent, suggesting that similar changes in the fatty acid composition took place in the phospholipids of mitochondrial membrane. Incorporation of phospholipids containing 13-MTD into membrane structure may impose a change in the membrane environment as is the case of death stimulus. The physicochemical properties of CLA are similar to those of unsaturated fatty acid, and may be more acceptable as membrane component. Thus the change in the membrane lipid profile elicited by incorporation of 13-MTD alternatively may affect the permeability of mitochondrial membrane, and result in the preceded release of AIF. The physicochemical properties of 13-MTD thereby may be a critical determinant to induce AIF translocation.

Majority of proapoptotic stimuli, including anticancer drug require a caspase dependent death pathway. It has been shown that AIF determines the chemoresistance of non-small-cell lung cancer cells [47]. In this case, activation of caspase cascade was insufficient to kill cancer cells, and an AIF-mediated death pathway played an important role for the induction of apoptosis [47]. Although it may be difficult to achieve mM concentration of 13-MTD in humans, present findings warrant further investigation to develop additional chemotherapeutic agent that is effective to kill the cancer cells resistant to the induction of apoptosis by conventional anticancer drug.

## Conclusion

In conclusion, we report that the 13-MTD was incorporated into glycerolipids of SKBR-3 cells with the preference for triglycerides rather than phospholipids, suggesting that incorporation of phospholipids containing 13-MTD into membrane structure influenced the membrane environment as are the case of death stimulus. Treatment with 13-MTD induced no change in the activity and expression of caspase 3. Furthermore, 13-MTD induced no mobilization of cellular calcium. However, 13-MTD altered the mitochondrial transmembrane potential and induced AIF translocation from mitochondrial to nuclear after 4 hrs of treatment. These results supported the view that incorporation of 13-MTD into cellular lipids triggered apoptosis via caspase-independent pathway.

## Materials and methods

### Chemicals

Enzyme phospholipase A_2 _was purchased from Sigma Chemicals Co.(Tokyo, Japan) and pancreatic lipase from Funakoshi(Tokyo, Japan). The primers were obtained from Hokkaido System Science Co., Ltd (Hokkaido, Japan). The RNA isolation kit was purchased from Wako (Osaka, Japan) and cDNA synthesis kit from Invitrogen(Tokyo, Japan). Other chemicals were all guaranteed grade, and obtained from domestic suppliers.

### Cell and tissue culture

Human breast cancer cell line SKBR-3 was purchased from Dainippon pharmaceuticals Co. (Osaka, Japan). Cells were maintained and sub cultured according to the supplier's recommendation. Culture medium for SKBR-3 was McCOY's 5A containing 10% FBS. Cells were cultured in a humidified atmosphere of 5% CO_2 _at 37°C

### Cell cytotoxicity assay

Test substances of free fatty acids were neutralized and dissolved in PBS containing 0.8% tween 80. Cell cytotoxicity titration curve was constructed with serial dilution of the test substances in a 96-well microplate. Cells seeded at density of 700 cells/well were incubated with serially diluted test substances, and the viable cell numbers were determined by MTS assay according to the manufacture's instruction (CellTiter^® ^AQueous Non-Radioactive Cell Proliferation Assay, Promega Co., Madison, USA). Cell cytotoxicity was thus expressed as the relative viability against control cells treated only with the vehicle solutions.

### Quantitation of apoptotic cells

SKBR-3 cells (1 × 10^4 ^cells/dish) were pre-incubated in the medium for 2 hours, and cultured further with fatty acid samples for 4 hours. At the end of the treatment, cells were washed with PBS and resuspended in 100 *μ*l of binding buffer containing Anexin V (MBL, Nagoya, Japan) and incubated for 30 min. Cells were washed with binding buffer, and apoptotic cells stained with Anexin V were counted with a fluorescence microscope. Data were expressed as percentages of apoptotic cells in the total cells.

### Lipid extraction and fractionation

SKBR-3 cells were seeded in 90 × 17 mm petridish at density of 1 × 10^4 ^/dish and grown to 80% confluence. Cells were subsequently incubated with 0.25 mM fatty acids dissolved in PBS containing 0.8% Tween 80 for 3, 6 and 24 h, respectively. After incubation, surface of the cultures were washed twice with PBS. Cells were scraped, centrifuged for 10 min at 700 × g and extracted with 1 ml of chloroform/methanol (2:1, by vol.) at 40°C for 60 min. The extract was split into chloroform and methanol water layer by addition of 0.2 ml water. Lipids partitioned into chloroform layer were concentrated and spotted onto HPTLC plates (Merck, Darmstadt, Germany) for a separation of triacylglycerol, (TG) and phospholipid (PL) fractions as described previously [3]. In the case of separation of phospholipids, the HPTLC plates were developed stepwise with chloroform/acetone/methanol (9:0.5:0.5, by vol.), chloroform/ethyl acetate/methanol/2-propanol/triethylamine/water (6.4: 1.5: 1.7: 0.05: 0.3: 0.2, by vol.). Phosphatidylethanolamine (PE) and phosphatidylcholine (PC) were identified by comparison of *R*_*f *_(retardation factor) value with authentic standards. TG and PL factions were extracted and methanolysed with methanolic hydrochloric acid as described elsewhere [14]. The fatty acid methyl esters produced were analyzed by a gas chromatograph (Model GC-2010; Shimadzu, Kyoto, Japan) with a capillary column (DB-FFAP column 0.25 mm ID × 30 m; J&W Scientific, CA, USA). Injector and detector temperatures were 210°C. The oven temperature was programmed as follows: the initial temperature was 50°C for 1 min, raised to 100°C at 10°C/min, thereafter to final temperature of 240°C at 5°C/min, and kept for 26 min. The chromatograms were analyzed by GC Solution Software (Shimadzu).

### Positional distribution of fatty acid

The method of enzymatic hydrolysis was used to analyze the positional distribution of fatty acid in PL and TG. PE and PC fractions were hydrolyzed with phospholipase A_2 _from bee venom (Sigma Chemicals Co.) About 1 mg of PE or PC in 1 ml diethyl ether was treated with 0.5 ml (850 U) phospholipase A_2 _dissolved in 1 M Tris buffer (pH 7.5) containing 4 mM calcium chloride. The enzyme reaction was monitored by examining the reaction products by HPTLC. Fractions of PE or PC were completely hydrolyzed to give rise to lyso PE or lyso PC, respectively. The released free fatty acid and lyso PE or lyso PC were analyzed for fatty acid compositions of sn-2 and sn-1, respectively.

In the case of TG, pancreatic lipase (Funakoshi, Japan) was used for the enzymatic hydrolysis. About 50 *μ*g of TG was mixed stepwise with 0.5 ml of 1 M Tris buffer (pH 8.0), 50 *μ*l of 2.2%calcium chloride and 0.125 ml of 0.05% cholic acid sodium salt. The mixture was incubated at 37°C for 1 min. Pancreatic lipase (40 *μ*l, 1 U/*μ*l) was added to this solution and shaken vigorously at 37°C for 1 h. The reaction was stopped by the addition of 0.5 ml of ethanol and 6 M HCl, and extracted with 4 ml of diethyl ether. The extract was washed with 1.5 ml of distilled water and dried over sodium sulphate. The reaction products, free fatty acid (FFA) and monoacylglycerol (MG), were separated by HPTLC with hexane/diethyl ether/acetic acid (5:5:0.1, by vol.), and analyzed for fatty acid composition of n-1,3 and n-2, respectively.

### Measurement of caspase activity

Cells were incubated with fatty acid sample for 2 or 4 hours and collected to measure caspase activity. Cells were lysed in buffer containing 10 mM Tris-HCl (pH 7.5), 10 mM Na_2_H_2_PO_4_/Na_2_HPO_4_, 130 mM NaCl, 1% Triton X-100 and 10 mM sodium pyrophosphate. The cell lysates (45 μl) were mixed with 5 *μ*l of fluorogenic caspases-3 substrate (500 *μ*M) DEVD-R110 (Roche Diagnostics, Mannheim, Germany) in 96-well micro titer plate according to the manufacturer's instruction. The plate was incubated at 37°C for 1 h, and then fluorescence was monitored with excitation and emission wavelengths at 485 and 535 nm, respectively.

### Real time PCR

Total RNA was extracted from 80% confluent SKBR-3 cells by RNA isolation kit, (Isogen, Wako, Osaka, Japan). A first strand cDNA synthesis was conducted by cDNA synthesis kit (Invitrogen, Japan). The sequences of the forward and reverse primers used were; caspases 3 (5'-GCCTGAGCAGAGACATGACTCA-3 and 5'-TCATCCACACATACCAGTGCGA-3), caspases 8 (5'-GATATATCCCGGATGAGGCTGAC-3 and 5'-TGACTGGATGTACCAGGTTCCC-3), P53 (5'-GGGATGTTTGGGAGATGTAAGAAATG-3 and 5'-GTGGGCCCCTACCTACCTAGAATG-3), AIF (5'-AGTAGTTTGCCCACAGTTGGTGTT-3 and 5'-TCACTCTCTGATCGGATACCAGTTC-3), Bad (5'-CAGTGACCTTCGCTCCACATC-3 and 5'-AAGGAGACAGCACGGATCCTC-3), Bax (5'-TCTGACGGCAACTTCAACTGG-3 and 5'-AGCCCATGATGGTTCTGATCA-3), Bcl-XL (5'-CCTCAGCTGCCTCACTTCCTA 3 and 5'-CCATAGCTGTTCCTGATAGCTCC-3), and GAPD (5'-TCTGGTAAAGTGGATATTGTTGCC-3 and 5'-CCTTCTTGATGTCATATTTGGC-3).

Each 20 *μ*l PCR reaction contained 0.5 U DNA polymerase (TaKaRa, Kyoto, Japan), 20 mM Tris-HCl (pH 8.4), 100 mM KCl, 0.1 mM EDTA, 1 mM DTT, 0.5% Tween 20, 0.5% Nonident P-40, 50% glycerol), 2.5 mM dNTP, 3 *μ*g cDNA, 10 pmol primers and 0.2 *μ*l of 1 × working solution of SYBR Green II (Takara, Shiga, Japan). The real time quantitative PCR was conducted in the thermocycler (iCycler, BioRad, Hercules, CA). The temperature program for real time analysis consisted of 30 cycles of 90 °C for 30 s, 58 °C for 30 s and final extension step at 72 °C for 30 s. Threshold cycle (C_T_) numbers were calculated during PCR amplification by using the iCycler data analysis software. The house-keeping GAPD genes were internal control to normalize the PCR for the amount of template cDNA. Relative change in gene expression was quantified essentially based on the 2^-ΔΔCT ^methods as described previously [15]. The 2^-ΔΔCT ^denotes the relative change of target gene expressed in sample-treated cells compared with that in reference cells [15]. Because the normalized concentration of cDNA template was used in this experiment, 2^-ΔCT ^represents the relative change of gene expression between treated and untreated cells. The equation that describes the relative change in threshold cycle is therefore

ΔC_T _= C_T _(sample)-C_T _(reference)

where C_T _is the threshold cycle reflecting the cycle number at which the fluorescence generated within a reaction process crosses the threshold level. In this study, sample cDNAs were serially diluted, and the mean C_T _and its 95% confidence interval were estimated by linear regression.

### Measurement of cellular calcium mobilization

SKBR-3 cells were seeded into black 96-well plate at 10^5 ^cells/well, and incubated for 1 day. The cells were washed twice with Hank's balanced salt solution (HBSS), and loaded with 4 μM Fluo 3-AM for 30 min in HBSS. The plates were washed with HBSS, transferred to microplate reader (Wallac 1420 Multilabel Counter, Parkin Elmer Co., Turka, Finland), and the basal fluorescence levels were recorded. Cells were loaded with 0.25 mM fatty acids samples, mixed for 30 s, and the increases in fluorescence were measured 25 times with 0.15 s intervals. Excitation and emission wavelength were 485 and 535 nm, respectively.

### Immunocytochemistry

For immunocytochemical analysis, cells grown on coverslips were fixed with 4% paraformaldehyde in PBS for 20 min, and washed twice with PBS. The cells were further permeabilized with 0.1% Triton X-100 in PBS for 20 min. After two washes with PBS, cells were incubated with diluted (1/200) rabbit anti-AIF (Chemicon International Co., CA, USA) in PBS containing 0.1% Tween 20(PBST) for 2 hours at room temperature. The cells were washed three times with PBST, and incubated with FITC-labeled goat anti-rabbit IgG (Biosource International Co., CA, USA) in PBST for 2 hours. After three washes with PBST, the cells were loaded with 1 μg/ml of Hoechst 33258 to stain DNA. Coverslips were then placed on a slide glass with cell-side down, and were covered with nail lacquer to prevent them from drying. The fluorescent images were visualized by conventional (Model BX41, Olympus Co., Tokyo, Japan) or confocal fluorescence microscopy (Radiance 2100, Bio-Rad Laboratories, Inc. Tokyo, Japan).

### Statistical analyses

Data are represented as mean ± SE except otherwise stated. The statistical significance was evaluated by Tukey-Kramer tests [16] or Student's t-test. The criterion for statistical significance was p <0.05.

## List of abbreviations

13-MTD, 13-methyltetradecanoic acid; BCFA, branched-chain fatty acid; C14:0, myristic acid; C18:0, stearic acid; HPTLC, high-performance thin layer chromatography; PL, phospholipid; TG, triglycerides; MG, monoacylglycerol; DG, diglyceride; PE, phosphatidylethanolamine; PC, phosphatidylcholine; FFA, free fatty acid; PBS, phosphate-buffered saline; FBS, fetal bovine serum, FA, fatty acid; PCR, polymerase chain reaction; IgG, immunoglobulin G; HBSS, Hanks' balanced salt solution; cDNA, complementary DNA; R_*f*_, retention factor; DMSO, dimethyl sulfoxide; DTT, dithiothreitol; RNA, ribonucleic acid; SE, standard error; SEM, standard error of the mean; TAG, triacylglycerol.

## Authors' contributions

WS and OH participated in study design, and drafted the manuscript. IH carried out the real time PCR. IM participated in immunochemical studies. TT and TY helped to draft the manuscript. All authors read and approved the final manuscript.
